# Fighting the flu in the tropics: The role of influenza vaccination in Southeast Asia

**DOI:** 10.5415/apallergy.0000000000000195

**Published:** 2025-03-17

**Authors:** Henry Sutanto, Alief Waitupu, Galih Januar Adytia, Deasy Fetarayani

**Affiliations:** 1 Internal Medicine Study Program, Department of Internal Medicine, Faculty of Medicine, Universitas Airlangga, Surabaya Indonesia; 2 Department of Internal Medicine, Dr. Soetomo General Academic Hospital, Surabaya, Indonesia; 3 Division of Allergy and Clinical Immunology, Department of Internal Medicine, Faculty of Medicine, Universitas Airlangga, Surabaya, Indonesia

**Keywords:** Influenza virus, public health surveillance, Southeast Asia, vaccine, virology

## Abstract

Influenza remains a significant public health concern globally, including in Southeast Asia, where unique epidemiological patterns and year-round virus circulation necessitate tailored vaccination strategies. This article briefly explores the historical milestones of influenza vaccine development, tracing its evolution from early inactivated vaccines to modern formulations. It also examines the annual inclusion of specific virus strains in vaccines, detailing the codename system for strain identification. In tropical regions like Southeast Asia, the need for influenza vaccination is debated due to continuous exposure; however, evidence supports its efficacy in reducing disease burden. It also discusses World Health Organization guidelines for optimal vaccination timing based on regional influenza activity and identifies target populations, including high-risk groups, and considerations for broader immunization efforts. Addressing these factors can enhance vaccination strategies and reduce influenza’s impact in Southeast Asia.

## 1. Introduction

Influenza is a significant global health concern, with a widespread impact on morbidity, mortality, and healthcare systems. Annually, influenza leads to severe illness and economic losses worldwide, particularly in regions with high population density and limited healthcare resources [1]. In Southeast Asia, influenza poses unique challenges due to year-round virus circulation influenced by tropical climates and varying levels of healthcare infrastructure [2]. The region’s dense population and high rates of poultry farming also make it a hotspot for the emergence of zoonotic influenza strains, such as H5N1, with pandemic potential [3]. Despite these risks, vaccine uptake and adherence to global vaccination guidelines remain low in the region, partly due to logistical challenges, funding issues, and low public awareness [4]. A systematic review focusing on Asian countries reported a median uptake of 14.9% among the general population and 37.3% among high-risk groups, which is significantly below the World Health Organization (WHO)’s target of 75% [2, 5]. Addressing these gaps is critical to reducing influenza’s impact in Southeast Asia. This article highlights the historical development of influenza vaccines, discusses the relevance of vaccination in tropical regions, and provides actionable recommendations for improving vaccination strategies.

## 2. Historical milestones of influenza vaccine development

**Figure 1.**
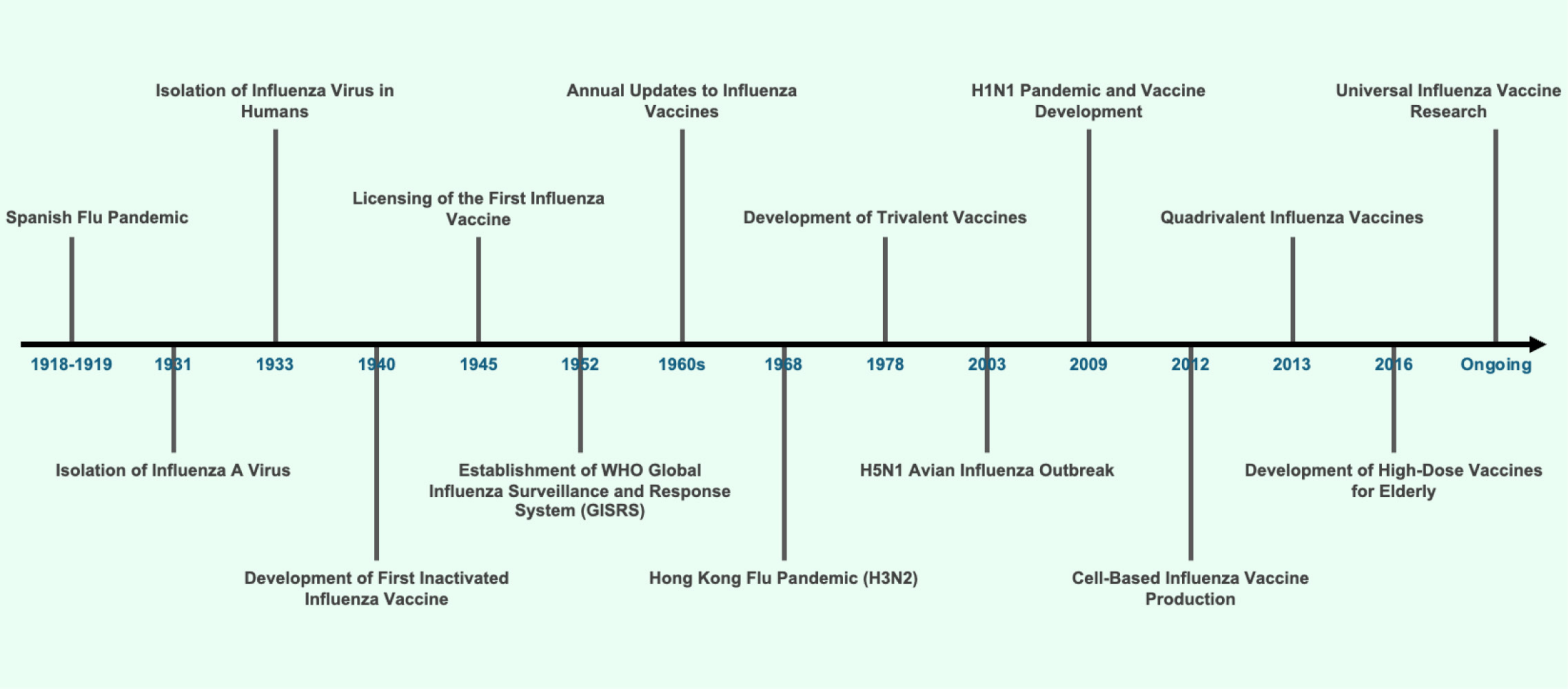
Timeline of influenza virus research and vaccine development. The timeline illustrates key milestones in influenza virus research and vaccine development, starting from the Spanish Flu pandemic (1918–1919) to ongoing efforts in universal influenza vaccine research.

The history of influenza vaccine development began with the discovery of the influenza virus in the early 20th century (Figure 1). The influenza A virus was first isolated from pigs in 1931 by Richard Shope, followed by the isolation of the virus from humans by Wilson Smith, Christopher Andrewes, and Patrick Laidlaw in 1933 [6, 7]. This discovery laid the foundation for understanding the virus’s structure and led to the first attempts at vaccine development. By the late 1940s, methods for growing the virus in embryonated chicken eggs were established, enabling mass production of vaccines. The initial influenza vaccines were inactivated formulations, which are still the most widely used today. The first licensed influenza vaccine was introduced during World War II to protect the United States (US) troops. Over the decades, advancements have included the development of live-attenuated vaccines and cell-based production methods, which improve the scalability and speed of vaccine manufacturing [8]. Recent innovations also include recombinant vaccines and the exploration of universal vaccines targeting conserved regions of the virus, aiming to provide broader and longer-lasting protection [1]. Global collaborations have been instrumental in advancing influenza surveillance and vaccine development. The WHO established the Global Influenza Surveillance and Response System (GISRS) in 1952 to monitor circulating strains and provide data for vaccine formulation. GISRS coordinates laboratories worldwide to detect emerging strains and update vaccine compositions biannually to match the most prevalent strains [9]. This system has been crucial for ensuring the efficacy of vaccines in controlling seasonal influenza and mitigating potential pandemics, such as the H1N1 pandemic in 2009.

## 3. Timeline of strains included in the vaccine

Influenza viruses are categorized into 4 main types: A, B, C, and D, with types A and B being most relevant to human disease and seasonal epidemics. Type A viruses are further divided into subtypes based on 2 surface proteins: hemagglutinin (HA) and neuraminidase (NA). Common subtypes include A(H1N1) and A(H3N2), which circulate widely among humans [10]. Influenza B viruses are not subdivided into subtypes but are divided into 2 lineages: B/Victoria and B/Yamagata. Each year, vaccines are formulated to target circulating strains from these categories, reflecting the dynamic nature of influenza viruses (Table 1). The WHO oversees the GISRS, which monitors influenza activity worldwide. Twice annually—once for the Northern Hemisphere and once for the Southern Hemisphere—GISRS experts convene to recommend strains to be included in the upcoming vaccines. The selection is based on extensive analysis of epidemiological data, antigenic properties, and genetic sequencing of circulating strains [11]. This ensures that vaccines match the most prevalent strains, providing the highest likelihood of protection. Each influenza vaccine strain is named using a standardized format: virus type (A or B), geographic origin, strain number, year of isolation, and subtype. For instance, A/Hong Kong/2671/2019 (H3N2) represents an influenza A virus isolated in Hong Kong, strain number 2671, identified in 2019, with H3N2 subtype. Similarly, B/Washington/02/2019 indicates an influenza B virus of the Washington lineage [1, 12]. These codenames help scientists and policymakers quickly identify the specific strains targeted by vaccines and ensure global consistency in tracking influenza viruses.

**Table 1.** Historical (egg-based) influenza vaccine strains in the southern hemisphere

Year	Vaccine strains included	Details
1978	A/Victoria/3/75 (H3N2) A/USSR/90/77 (H1N1) B/Hong Kong/8/73	First trivalent vaccine with 2 A strains and 1 B strain
1990	A/Shanghai/11/87 (H3N2) A/Singapore/6/86 (H1N1) B/Yamagata/16/88	Updated to include newly prevalent strains in both lineages
2000	A/Moscow/10/99 (H3N2) A/New Caledonia/20/99 (H1N1) B/Beijing/184/93	Continued inclusion of drifted H3N2 and stable B lineage
2010	A/California/7/2009 (H1N1) A/Perth/16/2009 (H3N2) B/Brisbane/60/2008	First inclusion of the 2009 H1N1 pandemic strain
2013	A/California/7/2009 (H1N1)pdm09 A/Victoria/361/2011 (H3N2) B/Wisconsin/1/2010 (B/Yamagata lineage) B/Brisbane/60/2008 (B/Victoria lineage)	Quadrivalent vaccines added a second B strain to expand coverage
2020	A/Brisbane/02/2018 (H1N1)pdm09 A/South Australia/34/2019 (H3N2) B/Washington/02/2019 (B/Victoria lineage) B/Phuket/3073/2013 (B/Yamagata lineage)	Updated strains targeting recent antigenic drift in both A and B viruses
2021	A/Victoria/2570/2019 (H1N1)pdm09 A/Hong Kong/2671/2019 (H3N2) B/Washington/02/2019 (B/Victoria lineage) B/Phuket/3073/2013 (B/Yamagata lineage)	Updated to include the most recent strains to match circulating viruses
2022	A/Victoria/2570/2019 (H1N1)pdm09 A/Darwin/9/2021 (H3N2) B/Austria/1359417/2021 (B/Victoria lineage) B/Phuket/3073/2013 (B/Yamagata lineage)	Adjusted to reflect the latest circulating strains, particularly the updated H3N2 component
2023	A/Sydney/5/2021 (H1N1)pdm09 A/Darwin/9/2021 (H3N2) B/Austria/1359417/2021 (B/Victoria lineage) B/Phuket/3073/2013 (B/Yamagata lineage)	Maintained quadrivalent format to address circulating A and B strains globally
2024	A/Victoria/4897/2022 (H1N1)pdm09 A/Thailand/8/2022 (H3N2) B/Austria/1359417/2021 (B/Victoria lineage) B/Phuket/3073/2013 (B/Yamagata lineage)	Continued inclusion of updated strains to match circulating viruses, with emphasis on recent H1N1 and H3N2 variants
2025	A/Victoria/4897/2022 (H1N1)pdm09 A/Croatia/10136RV/2023 (H3N2) B/Austria/1359417/2021 (B/Victoria lineage) B/Phuket/3073/2013 (B/Yamagata lineage)	Continued inclusion of updated strains to match circulating viruses

## 4. Progress on formulations and technologies of the influenza vaccines

### 4.1. Inactivated, live-attenuated, recombinant, and virus-like particle formulations

Influenza vaccines come in various formulations, each with distinct immunogenicity profiles, mechanisms of action, and protective efficacy. The four primary types: inactivated influenza vaccines (IIVs), live-attenuated influenza vaccines (LAIVs), recombinant influenza vaccines (RIVs), and virus-like particle (VLP) vaccines, offer different advantages and limitations based on their antigen presentation and immune activation mechanisms. Conventional influenza vaccines, primarily IIV or LAIV formulations, offer variable efficacy, especially against antigenically drifted strains. IIVs, also known as conventional flu shots, consist of chemically inactivated whole or split influenza viruses that induce an immune response primarily through humoral immunity. These vaccines elicit robust antibody production against HA and NA, leading to protection against homologous strains [13]. However, IIVs are less effective against antigenically drifted strains and do not elicit strong mucosal or cellular immunity. In a comparative study, IIVs provided better serological responses compared with LAIVs, particularly in elderly individuals [14]. LAIVs contain weakened influenza viruses that replicate in the nasal mucosa but do not cause disease. They induce both mucosal and systemic immune responses, making them more effective at generating long-term immunity compared with IIVs. Studies show that LAIVs elicit stronger T-cell responses and promote local secretory IgA production, enhancing protection against antigenic drift [15]. However, LAIV efficacy has shown variability in older populations and may be less effective against type B influenza viruses [13].

RIVs use recombinant technology to express influenza HA proteins without the need for egg-based production. This method allows for rapid scalability and avoids issues related to egg adaptations. Studies have demonstrated that recombinant HA and NA-based vaccines induce strong humoral and cellular immunity, providing broader protection compared with IIVs and LAIVs [16]. Additionally, RIVs have been shown to elicit cross-protective immunity against antigenically drifted influenza strains [17]. However, these vaccines primarily induce a humoral response and may not be as effective at stimulating mucosal immunity compared with LAIVs. VLP vaccines mimic the structure of influenza viruses without containing the genetic material, making them safer and highly immunogenic. These vaccines elicit robust immune responses, including strong T-cell activation and mucosal immunity. Compared with IIVs and RIVs, VLPs generate broader immune responses against antigenically distinct strains [18]. In animal models, VLP vaccines provided superior cross-protection against heterologous strains, suggesting their potential as universal flu vaccines [19]. Overall, while IIVs provide reliable protection through robust antibody responses, they lack mucosal immunity and T-cell activation. LAIVs induce broader immunity but exhibit variable efficacy in different age groups. RIVs offer rapid production and enhanced humoral immunity but require adjuvants to improve cell-mediated responses. VLPs, on the other hand, provide strong and broad immunity but remain in early clinical development.

### 4.2. Trivalent vs quadrivalent formulations

Traditional trivalent influenza vaccines (TIVs) contain 2 influenza A strains (H1N1 and H3N2) and 1 influenza B strain. However, since 2 antigenically distinct B lineages (Victoria and Yamagata) co-circulate, mismatches between the circulating and vaccine B strains can occur, reducing vaccine effectiveness. Quadrivalent influenza vaccines (QIVs) include both B lineages, potentially providing broader protection against influenza B. Several studies have shown that QIVs induce a broader immune response than TIVs without compromising immunogenicity against shared strains. A phase III clinical trial comparing QIV with 2 different TIV formulations demonstrated that QIV met noninferiority criteria for common strains while providing superior immune responses against the additional B strain [20]. Similarly, a meta-analysis found that QIV offered superior protection against both B lineages, with a significant increase in seroconversion rates compared with TIV [21]. Clinical effectiveness studies also favor QIVs, particularly in seasons with high influenza B activity. A historical cohort study conducted over 2 influenza seasons showed that QIV recipients had lower hospitalization and emergency department visit rates than TIV recipients [22]. Furthermore, in children, QIV demonstrated superior immune responses against the non-TIV B lineage, reducing the likelihood of mismatches [23].

Both QIV and TIV have comparable safety profiles, with no significant differences in adverse events. Local reactions, such as injection-site pain, were slightly more frequent with QIV, but systemic reactions (eg, fever, fatigue) were similar between vaccine groups [24]. Importantly, no vaccine-related serious adverse events were reported in multiple randomized controlled trials. In pediatric populations, the addition of the second B lineage did not increase reactogenicity, making QIV a suitable alternative to TIV for young children [25]. Economic analyses suggest that the broader protection offered by QIV may lead to significant cost savings. A Monte Carlo simulation model estimated that switching from TIV to QIV could result in a median societal cost savings of $3.1 billion over 10 influenza seasons in the US [26]. This is due to reductions in influenza-related hospitalizations, outpatient visits, and lost productivity. Another study demonstrated that QIV could reduce influenza B-related cases by 23% compared with TIV, further highlighting its economic and public health benefits [27].

### 4.3. Cell-based vs egg-based influenza vaccines

Influenza vaccines have traditionally been manufactured using embryonated chicken eggs, a process that has been in place for decades. However, cell-based vaccine production has emerged as an alternative, offering potential advantages in scalability, efficiency, and antigenic fidelity. Cell-based influenza vaccines are produced in mammalian cell cultures, such as Madin-Darby canine kidney cells, rather than in eggs. One of the major concerns with egg-based vaccines is the potential for adaptive mutations that alter antigenicity, leading to mismatches between the vaccine strain and circulating influenza viruses [28]. Several studies have suggested that cell-based vaccines may provide better protection against antigenic drift, particularly for influenza A(H3N2), a strain that frequently undergoes significant changes [29]. A comparative study conducted during the 2017 to 2018 influenza season found that cell-based vaccines provided better protection against influenza A(H3N2) than egg-based vaccines, with a relative vaccine effectiveness of 39.6% for influenza B and 8.0% for influenza A [29]. Similarly, a meta-analysis of real-world data concluded that cell-based vaccines were more effective than egg-based vaccines across multiple influenza seasons [30]. Studies indicate that cell-based and egg-based vaccines have comparable safety profiles. A phase III clinical trial found no significant difference in adverse events between the 2 vaccine types, although mild local reactions such as injection-site pain were slightly more common with cell-based vaccines [31]. A retrospective cohort study assessing adverse events in England also reported no major differences in safety outcomes between cell-based and egg-based vaccines [32].

One of the major advantages of cell-based vaccine production is its flexibility and scalability. Unlike egg-based production, which is dependent on the availability of embryonated eggs, cell-based production can be rapidly scaled up in response to pandemics [33]. Furthermore, cell-based vaccines are free from egg-related adaptations, potentially improving antigenic match to circulating influenza strains. In general, cell-based influenza vaccines offer advantages over egg-based vaccines in terms of antigenic fidelity, effectiveness against antigenic drift, and pandemic preparedness. While both vaccine types have comparable safety profiles, cell-based vaccines have demonstrated superior immunogenicity, particularly for influenza A(H3N2).

### 4.4. Standard-dose vs high-dose influenza vaccine

Standard-dose influenza vaccines (SDVs) contain 15 µg of HA per strain, whereas high-dose influenza vaccines (HDVs) contain 60 µg per strain. HDVs were developed to enhance immune responses in populations with weaker immunity, such as individuals aged 65 years and older. Several studies have demonstrated that HDVs elicit stronger immune responses than SDVs. A large randomized controlled trial found that HDVs induced significantly higher hemagglutination inhibition (HAI) titers and seroprotection rates compared with SDVs [34]. Additionally, a systematic review and meta-analysis confirmed that HDVs provide enhanced immunogenicity and seroconversion rates in older adults [35]. In terms of clinical effectiveness, HDVs have shown superior protection against laboratory-confirmed influenza and its complications. A Medicare cohort study reported that high-dose vaccines were 24% more effective in preventing postinfluenza deaths compared with SDVs [36]. Another study found that HDVs reduced hospital admissions due to respiratory illnesses by 17.7%, particularly in reducing the risk of serious pneumonia [37].

The efficacy of HDVs varies by influenza season and circulating strain. During the 2012 to 2013 influenza season, when H3N2 was the predominant strain, HDVs were 36.4% more effective than SDVs in reducing mortality. However, in the following season (2013–2014), the advantage of HDVs was less pronounced, with only a 2.5% increased effectiveness [36]. This suggests that HDVs may be particularly beneficial when vaccine–virus mismatch or antigenic drift occurs. Both HDVs and SDVs have similar safety profiles, with no significant differences in severe adverse events. A clinical trial found that rates of hospitalization and mortality were comparable between HDV and SDV recipients [34]. However, HDVs are associated with a slightly higher incidence of mild local reactions, such as pain and redness at the injection site, which are generally well tolerated. Despite their higher cost, HDVs may offer economic benefits due to reduced hospitalization rates and healthcare utilization. A cost-effectiveness analysis estimated that switching from SDVs to HDVs could save millions in medical costs annually by preventing severe influenza cases [38].

### 4.5. Novel route of administration: intradermal influenza vaccine

Intradermal (ID) influenza vaccines have emerged as an alternative to traditional intramuscular (IM) administration, offering potential benefits such as dose sparing, enhanced immune responses, and better accessibility for mass immunization. The unique immunological environment of the dermis, which is rich in antigen-presenting cells (APCs), contributes to the effectiveness of ID vaccines. The dermis contains a high density of professional APCs, including Langerhans cells and dermal dendritic cells, which play a crucial role in antigen uptake, processing, and presentation to naive T cells. This rich network of immune cells allows for efficient activation of both humoral and cellular immune responses, often requiring a lower antigen dose than conventional IM vaccines [39]. Studies have demonstrated that ID influenza vaccination induces comparable or superior antibody responses with a fraction of the antigen dose, which is particularly valuable during vaccine shortages. ID influenza vaccines have been shown to elicit strong humoral immunity, characterized by the production of HAI antibodies. These responses are often comparable to or exceed those generated by IM vaccines. In a randomized trial, elderly recipients of an ID influenza vaccine exhibited significantly higher geometric mean titers compared with IM vaccine recipients [40]. Additionally, ID administration can enhance cross-protective immunity by generating broader and more diverse antibody responses, including responses to heterologous strains [41]. Beyond humoral immunity, ID vaccination also enhances cellular immunity by stimulating CD4^+^ T-helper cells and cytotoxic CD8^+^ T cells. Studies have demonstrated that ID vaccines induce stronger T-cell responses, which contribute to long-term immunity and better protection against antigenic drift [42].

ID vaccination is particularly beneficial for elderly and immunocompromised individuals, who often exhibit diminished immune responses to traditional IM influenza vaccines. A study comparing ID and IM vaccines in older adults found that ID vaccination elicited superior seroprotection and seroconversion rates [43]. Additionally, ID vaccination has been evaluated in immunocompromised patients, such as those undergoing hematopoietic stem cell transplantation or receiving anti-tumor necrosis factor (TNF) therapy, with findings indicating comparable efficacy to IM vaccination [44]. Efforts to further improve ID vaccine efficacy have explored the use of adjuvants such as imiquimod, a TLR7 agonist, which has been shown to significantly enhance and prolong immunogenicity when applied topically before ID vaccination [42]. Other adjuvants, such as virosomal formulations, have also demonstrated the ability to boost immune responses while maintaining a favorable safety profile.

### 4.6. Other emerging influenza vaccine technologies

Emerging vaccine technologies aim to enhance immunogenicity, durability, and breadth of protection by leveraging novel adjuvants, delivery platforms, and antigenic targets. Mucosal vaccines, particularly intranasal formulations, have gained attention due to their ability to elicit both systemic and mucosal immunity. These vaccines induce robust local immune responses, including secretory IgA production, which provides a first-line defense at the site of infection. Intranasal administration of LAIVs has shown superior mucosal and systemic humoral responses compared with parenteral inactivated vaccines [45]. However, safety concerns with live strains have driven research toward alternative mucosal vaccine strategies using adjuvanted or VLP formulations. In addition to intranasal vaccines, dry powder and aerosol formulations have emerged as viable alternatives to conventional injection-based immunization. Aerosolized vaccines provide direct access to the respiratory tract, triggering robust mucosal immune responses. Studies have demonstrated that spray-dried lipid formulations can significantly enhance immunogenicity and reduce the need for injectable vaccines [46]. Similarly, bioadhesive intranasal delivery systems utilizing microspheres have been shown to elicit strong mucosal and systemic immune responses [47]. Another vaccine technology aims to address the limitations of current influenza vaccines, which require annual reformulation due to antigenic drift and shift. Universal vaccine strategies seek to overcome this challenge by targeting conserved viral components, such as the HA stalk domain or the matrix protein M2. A chimeric HA-based vaccine has been shown to elicit broadly cross-reactive antibodies with long-lasting immunity [48]. Similarly, a TLR5 ligand-conjugated M2e-based vaccine has demonstrated potent immunogenicity and protection across multiple influenza strains [49].

Adjuvants are critical for improving the efficacy of both conventional and novel influenza vaccines. Oil-in-water emulsions, such as AS03 and MF59, enhance antigen presentation and immune response durability. AS03, in particular, has been shown to induce a proinflammatory environment by engaging the IRE1α stress sensor, thereby amplifying T follicular helper cell responses and improving antibody avidity [50]. Other promising adjuvants include CpG oligonucleotides, Toll-like receptor agonists, and chitosan-based adjuvants, which have demonstrated the ability to enhance T- and B-cell responses [51].

## 5. Relevance of influenza vaccination in tropical regions

Influenza epidemiology in tropical climates is distinct from temperate regions, as the virus exhibits year-round activity with occasional peaks during rainy or humid seasons. Unlike temperate zones, which experience predictable winter epidemics, tropical areas demonstrate continuous influenza virus circulation due to favorable climatic conditions such as high humidity and temperature. Studies have shown that year-round influenza activity is more prevalent in tropical countries compared with temperate ones, where epidemic patterns dominate [52]. Additionally, the lack of well-defined seasons in tropical areas leads to overlapping epidemic peaks, complicating predictions and intervention strategies [53]. Vaccination in tropical regions is critical to reducing the disease burden caused by influenza, which frequently manifests as respiratory infections and pneumonia. Studies emphasize that influenza vaccines can effectively reduce morbidity and mortality in these regions. For example, data from Southeast Asia highlight that timely vaccination campaigns, particularly before rainy seasons, can mitigate peak influenza activity and prevent significant public health burdens [54]. Additionally, tropical surveillance studies have shown that influenza vaccination reduces childhood pneumonia and respiratory infections, which are more prevalent due to the persistent virus circulation in tropical settings [55]. Yet, Southeast Asia faces several public health challenges regarding influenza vaccination. These include inadequate surveillance systems, logistical difficulties in vaccine distribution, and low public awareness of vaccination benefits. The region’s diverse epidemiological patterns require tailored vaccination strategies to align with local virus circulation trends. For instance, vaccination timing recommendations remain unclear for countries such as Laos, Cambodia, and Thailand due to complex and inconsistent seasonality [56]. Despite these challenges, increasing regional collaboration and year-round surveillance efforts are pivotal to improving vaccine coverage and preparedness for potential pandemic strains [57].

## 6. Barrier to vaccination in Southeast Asia region

### 6.1. Timing of vaccination

The timing of influenza vaccination varies widely across regions due to the diverse patterns of influenza activity influenced by climatic and geographical factors. In temperate regions, influenza activity peaks during the winter months, leading to a clear seasonal vaccination schedule. However, in tropical and subtropical regions, influenza circulation is less predictable, often occurring year-round or with peaks during rainy seasons. For example, countries closer to the equator, such as Singapore and Indonesia, experience continuous influenza activity without discrete peaks, whereas nations such as Thailand and Vietnam show distinct surges in influenza activity between July and October, aligning with the monsoon season [56]. This variability necessitates tailored vaccination strategies to maximize the vaccine’s effectiveness within local contexts. Regions such as Southeast Asia must navigate complex influenza patterns, making it challenging to determine the optimal vaccination period. Surveillance data are critical to understand these patterns and guiding vaccination campaigns accordingly [1]. To address the complexities of influenza activity in tropical regions, the WHO has provided specific recommendations for vaccination timing. In Southeast Asia, where influenza activity peaks earlier than in temperate regions, the WHO advises vaccination campaigns to occur between April and June. This timing ensures that vaccine-induced immunity is highest during the critical months of influenza circulation, particularly from July to October. Countries such as Thailand, Cambodia, and Vietnam have been encouraged to adopt tailored vaccination policies to align with their unique epidemiological patterns. For regions with year-round circulation, such as Malaysia and Indonesia, the WHO suggests that vaccination strategies should be flexible, allowing for periodic or even biannual campaigns to sustain immunity throughout the year [56]. These recommendations highlight the importance of leveraging regional data and climatic insights to design effective vaccination strategies.

### 6.2. Logistic, funding, and infrastructure issues in the region

One of the primary logistical barriers to influenza vaccination in Southeast Asia is the complexity of vaccine distribution. Many countries in the region face challenges related to transportation infrastructure, particularly in rural and remote areas. Limited cold chain capacity affects vaccine storage and delivery, leading to vaccine wastage and reduced availability in underserved regions [1]. Additionally, the reliance on imported vaccines and fluctuating supply chains results in periodic shortages, further complicating vaccination efforts. Furthermore, public-sector influenza vaccination programs in Southeast Asia are often underfunded, with limited financial resources allocated to vaccine procurement and administration. A survey of influenza vaccine policies found that only 5 countries in the region (ie, Indonesia, Malaysia, Singapore, Thailand, and Vietnam) had official influenza vaccination guidelines, and even fewer adhered to global recommendations [4]. In many countries, vaccination remains largely a private-sector initiative, with limited government-subsidized programs, making it inaccessible to lower-income populations. A shortage of trained healthcare workers to administer vaccines presents another challenge in the region. Many healthcare facilities, particularly in rural areas, lack the personnel needed to conduct large-scale vaccination programs. Additionally, vaccine hesitancy among healthcare workers, due to concerns about vaccine efficacy and safety, further reduces vaccination uptake [5]. Addressing these workforce limitations requires investment in training and awareness programs to enhance vaccine confidence and delivery capacity. On the whole, logistical challenges remain a significant barrier to influenza vaccination in Southeast Asia. Supply chain issues, seasonal variability, insufficient government funding, and a limited healthcare workforce all contribute to low vaccine coverage in the region. Strengthening cold chain infrastructure, aligning vaccination schedules with regional epidemiology, expanding government-funded programs, and investing in healthcare workforce training are essential steps toward improving vaccine accessibility and coverage.

## 7. Target populations for influenza vaccination

Influenza vaccination efforts should prioritize high-risk groups due to their increased susceptibility to severe outcomes from influenza infection. Pregnant women are among the most vulnerable, as influenza during pregnancy is associated with higher risks of hospitalization and complications for both mother and child. Vaccination during pregnancy not only protects the mother but also provides passive immunity to the newborn, a crucial benefit given the limited vaccination options for infants [58]. In Singapore, a study found that coverage among pregnant women to date was less than 10% [59]. Similarly, in Thailand, despite a national policy offering free influenza vaccination to pregnant women in their second and third trimesters, only 25% of physicians in antenatal clinics routinely recommend the vaccine [60]. The elderly population also remains a critical focus, as age-related immune decline (immunosenescence) makes them more susceptible to severe influenza outcomes, including pneumonia and cardiovascular complications. Strategies like biannual vaccination have been explored to enhance immunity among older adults in tropical regions, where year-round influenza circulation occurs [61]. In Singapore, a 2013 study reported that vaccination rates increased from 14.1% in individuals aged 50 to 54 years to 16.4% in those aged 65 to 69 years, with a slight decrease to 13.4% in the 70 to 74 age group, and the highest uptake of 22.4% observed in those aged 80 years and above [62]. In Thailand, despite the availability of free influenza vaccinations for individuals aged 65 years and older, uptake remains suboptimal [63]. A broader review indicated that overall seasonal influenza vaccination coverage was less than 1% in most parts of Asia, highlighting the significant gap in immunization among the elderly population in the region [64]. Young children, particularly those under 5 years of age, are another high-risk group due to their immature immune systems and higher likelihood of complications, including secondary bacterial infections. Influenza vaccination has been shown to significantly reduce respiratory illnesses and hospitalizations in this group [65]. In Thailand, studies have reported vaccination rates of approximately 1% among children aged 6 to 35 months in 2011 [66]. Another study indicated that nationwide coverage among Thai children aged 6 months to 2 years was 3% in 2010, 1% in 2012, and 2% in 2015 [67]. Healthcare workers are also prioritized because they are at the frontline of patient care and can act as vectors for influenza transmission, putting both themselves and their patients at risk. Vaccination among healthcare workers not only safeguards their health but also contributes to herd immunity within healthcare settings [68]. In Bangladesh, a study reported an overall vaccination uptake of 13.8% among healthcare workers, with higher rates in public hospitals (14.4%) compared with private hospitals (10.0%). Nurses had the highest coverage at 20.0%, followed by physicians at 13.5%, while cleaning staff had the lowest at 2.2% [69]. In Vietnam, research indicated that only a small proportion of medical students and healthcare workers demonstrated a high level of knowledge and positive attitudes toward influenza vaccination, suggesting low uptake rates [70]. Individuals with chronic illnesses, such as diabetes, asthma, or cardiovascular conditions, also face a higher likelihood of severe influenza complications and are therefore a key focus of vaccination campaigns.

Immunocompromised individuals, including those with primary and secondary immunodeficiencies, are at heightened risk of severe influenza-related complications. Despite being a key target group for vaccination, these individuals often exhibit suboptimal immune responses to standard influenza vaccines. Given these challenges, expert groups have established tailored recommendations to optimize protection while ensuring safety. Several international health organizations, including the Advisory Committee on Immunization Practices, the European Society of Clinical Microbiology and Infectious Diseases, and the Infectious Diseases Society of America, strongly recommend annual influenza vaccination for immunocompromised individuals [71]. The preferred vaccines for this population include IIVs and RIVs, as these formulations avoid the risks associated with live virus replication. High-dose or adjuvanted vaccines have been suggested as a means to enhance immune responses in immunocompromised patients. The high-dose trivalent inactivated vaccine (IIV3-HD) has demonstrated stronger humoral responses in transplant recipients, patients receiving immunosuppressive therapy, and individuals with HIV [72]. Additional strategies to improve immunogenicity include booster doses, ID vaccination, and temporary discontinuation of immunosuppressive therapy in select cases. While IIVs are generally safe for immunocompromised patients, LAIVs are contraindicated due to the potential risk of uncontrolled viral replication and vaccine-associated influenza infection [73].

Key precautions for various immunocompromised populations include: HIV-infected individuals should receive annual influenza vaccination with IIVs, as LAIVs pose a higher risk, particularly for those with CD4 counts below 200 cells/mm³ [71]. Solid organ and hematopoietic stem cell transplant recipients are advised to receive IIVs at least 6 months posttransplant to ensure optimal immune system recovery, with booster vaccinations recommended for those on high-dose immunosuppression [74]. Patients undergoing biologic therapy, such as those on TNF inhibitors or rituximab, may experience reduced vaccine efficacy, necessitating higher antigen doses or booster shots. Some expert guidelines suggest timing vaccination just before the next treatment cycle to maximize immune response [75]. Similarly, patients with autoimmune diseases receiving immunosuppressive therapies, including corticosteroids and methotrexate, may exhibit diminished vaccine responses, making high-dose or adjuvanted influenza vaccines preferable for enhanced protection [72]. Despite clear recommendations, influenza vaccine uptake remains suboptimal in immunocompromised populations. A large cohort study in France found that only 59% of immunocompromised patients received the influenza vaccine, highlighting the need for targeted educational campaigns and improved provider recommendations [75]. Additionally, some physicians hesitate to vaccinate immunocompromised patients due to concerns about vaccine efficacy and potential disease exacerbation.

In addition to targeting high-risk groups, there is growing consideration for broader influenza vaccination strategies in Southeast Asia. The region’s year-round virus circulation and dense population dynamics amplify the risk of widespread outbreaks, prompting discussions about mass vaccination campaigns. Mass vaccination can achieve indirect benefits, such as reducing community-level transmission and protecting vulnerable populations who may not respond robustly to vaccines, like the elderly. Studies in tropical settings have demonstrated that vaccinating younger, high-contact age groups, such as schoolchildren, can significantly reduce influenza mortality through herd immunity. Southeast Asia faces unique challenges in implementing mass vaccination strategies, including resource constraints and logistical difficulties. However, models suggest that even limited vaccine supplies can achieve substantial mortality reductions if optimally allocated to high-contact groups and extended to broader populations [76]. To boost public awareness and confidence in influenza vaccination, governments should strengthen national policies to ensure vaccines are widely available, particularly for high-risk groups, and implement mandates for healthcare workers and vulnerable populations. Community engagement through culturally relevant education programs is essential to address vaccine hesitancy, with messaging that highlights vaccine benefits, safety, and influenza risks while leveraging trusted community leaders and religious organizations for credibility. Healthcare workers, who play a crucial role in shaping public perception, should receive targeted training and promotion to encourage their participation in immunization campaigns [5]. Additionally, governments should provide free or subsidized vaccines to low-income groups, remove financial barriers, and introduce incentives such as workplace vaccination programs and insurance benefits to encourage uptake. Enhanced surveillance and data transparency are also critical, as improved epidemiological tracking can inform optimal vaccination timing and transparent reporting on vaccine effectiveness can build trust among the public [1]. Finally, integrating influenza vaccination into existing public health programs, such as childhood vaccinations or COVID-19 boosters, can streamline access and increase coverage while minimizing operational costs [77]. Considering the epidemiological characteristics of influenza in Southeast Asia, mass vaccination strategies could complement targeted approaches, offering broader community protection and potentially reducing the region’s significant burden of influenza-related morbidity and mortality.

## 8. Conclusion

Influenza vaccination plays a critical role in protecting public health in Southeast Asia, a region where year-round virus circulation and dense populations heighten the risk of outbreaks and severe disease. Prioritizing high-risk groups such as pregnant women, the elderly, young children, immunocompromised individuals, and healthcare workers, while exploring mass vaccination strategies, can significantly reduce the burden of influenza-related illness and mortality. However, achieving these goals requires addressing barriers to vaccination, including limited coverage and public awareness. A concerted effort to expand access, optimize vaccination timing, and educate communities about the benefits of immunization is essential to improving health outcomes and building resilience against influenza in the region.

## Conflicts of interest

The authors have no financial conflicts of interest.

## Author contributions

HS: conception of study, writing of manuscript, visualization, revisions, and approval of final draft. AW and GJA: writing of manuscript, revisions, and approval of final draft. DF: writing of manuscript, revisions, supervision, and approval of final draft.
